# Effect of probiotic supplementation on in-hospital mortality in patients with acute myocardial infarction: a study protocol for an open-label, randomized, controlled, superiority clinical trial

**DOI:** 10.1186/s13063-023-07443-5

**Published:** 2023-06-24

**Authors:** Yequn Chen, Rongbing Chen, Xin Wang, Yan Zhou, Liekai Hong, Nianling Xiong, Jinxiu Zhu, Shu Ye, Xuerui Tan

**Affiliations:** 1 The First Affiliated Hospital of Shantou University Medical College, Shantou, 515041 Guangdong China; 2 Clinical Research Center, The First Affiliated Hospital of Shantou University Medical College (SUMC), Shantou, China; 3 Shantou University Medical College, Shantou, China; 4 Chaonan Minsheng Hospital, Shantou, China; 5 Department of Cardiovascular Sciences and NIHR Leicester Biomedical Research Centre, University of Leicester, Leicester, UK

**Keywords:** Probiotics, Acute myocardial infarction, Major adverse cardiovascular events (MACE), Mortality, Randomized controlled trial

## Abstract

**Background:**

Recent studies have demonstrated a correlation between intestinal flora and the severity of myocardial infarction as well as post-myocardial infarction repair. However, few studies have investigated whether probiotics reduce mortality and improve cardiovascular outcomes in patients with acute myocardial infarction. In this study, we will conduct a randomized controlled trial (RCT) to evaluate the effect of probiotics on in-hospital mortality and the incidence of major adverse cardiovascular events (MACE) in patients with acute myocardial infarction (AMI).

**Methods:**

This is an open-label, randomized, controlled, superiority clinical trial involving 2594 adult patients who were diagnosed with acute myocardial infarction. Patients will be randomized to (1) receive bifidobacteria triple viable capsule (*Bifidobacterium longum*, *Lactobacillus acidophilu*s, and *Enterococcus faecalis*) 840 mg, twice a day, plus standard treatment strategy during the hospital stay, for a maximum of 30 days, or (2) receive the standard treatment strategy and will not take the bifidobacterium triple live capsule. The primary outcome was in-hospital all-cause mortality.

**Discussion:**

The purpose of this clinical trial is to determine whether probiotics can reduce in-hospital mortality and improve prognosis in patients with AMI, and the results will provide evidence for probiotics as a complementary treatment for AMI.

**Trial registration:**

Chinese Clinical Trials Registry ChiCTR2000038797. Registered on 2 October 2020.

**Supplementary Information:**

The online version contains supplementary material available at 10.1186/s13063-023-07443-5.

## Background

Acute myocardial infarction (AMI) is a severe form of coronary heart disease, and its high morbidity and mortality pose the main threat to human life and health. The incidence of adverse cardiovascular events after AMI, such as heart failure and arrhythmias, is associated with a substantially higher risk of death [1, 2]. In recent decades, with the rise of reperfusion therapy and the standardization of treatment for AMI, the mortality rate has decreased significantly. However, a significant inpatient mortality rate remains and can be as high as 8.9% [3], and the incidence of heart failure after acute myocardial infarction during hospitalization ranged from 40.2 to 40.5% [4]. Therefore, the treatment of acute myocardial infarction needs to be further optimized.

Recent studies have shown an important link between intestinal flora and the severity of myocardial infarction and heart repair after myocardial infarction [5, 6]. Intestinal disorders and bacterial translocations play an important role in the development of myocardial infarction and in cardiovascular events after infarction [7]. Patients with acute coronary syndrome (ACS) had typical intestinal microflora disorders, which were manifested as increased harmful bacteria and decreased beneficial bacteria [8]. Many animal studies have confirmed that probiotics can reduce the size of myocardial infarction and reduce cardiac hypertrophy [5, 9]. Several clinical trials have also reported that probiotics can reduce ventricular remodeling after MI and improve ventricular systolic function in patients with heart failure [10, 11], which imply that probiotic supplementation may improve the prognosis of acute myocardial infarction. However, it has not been reported whether probiotics can reduce the incidence of adverse cardiovascular events and mortality in patients with MI in the hospital. In this study, we will evaluate the effect of probiotics on in-hospital mortality and the incidence of major adverse cardiovascular events (MACE) in patients with AMI to understand whether probiotics have a protective effect on patients with acute myocardial infarction through a randomized controlled trial (RCT).

## Methods

### Study design

This prospective trial will be a single-center, open-label, randomized, controlled, superiority clinical trial. A total of 2594 patients with AMI (both acute ST-segment elevation and non-ST-segment elevation myocardial infarction) will be recruited and randomized in a 1:1 fashion to one of the two treatment arms from October 2020 through October 2025. The protocol was designed according to the Standard Protocol Items: Recommendations for Interventional Trials (SPIRIT) 2013 Statement [12]. The protocol was designed in accordance with the SPIRIT 2013 Statement (Standard Protocol Items: Recommendations for Interventional Trials). The SPIRIT Checklist for this protocol is detailed in the Supplementary Material, Additional file 2.

### Ethics issues

Ethical approval for the study has been obtained from the Committee of Ethics of the First Affiliated Hospital of Shantou University Medical College (Number: B-2020–165-FS).

### Study hypothesis and objectives

Studies have shown that patients with AMI have certain intestinal flora disorder and impaired intestinal barrier function [8]. When myocardial infarction occurs, intestinal bacteria and metabolites translocate into the systemic circulation, causing metabolic endotoxemia and stimulating systemic low-grade inflammation, thereby affecting the repair of the heart after myocardial infarction and the occurrence of adverse time after myocardial infarction [13]. Therefore, we hypothesized that early probiotic supplementation after AMI could have a positive effect on the heart by repairing the gut barrier, reducing endotoxemia and systemic inflammation.

The primary objective of this trial is to determine the effect of probiotics on in-hospital mortality of AMI patients. The secondary objective is to investigate whether probiotics can reduce the incidence of major adverse cardiovascular events (including heart failure, malignant arrhythmias, cardiogenic shock, myocardial infarction-related mechanical complications, and recurrent myocardial infarction) and improve ventricular systolic function in patients with AMI; echocardiography will be measured at baseline and after treatment.

### Population

Patients will be recruited from the First Affiliated Hospital of Shantou University Medical College, 3000 beds of teaching hospital in Guangdong, China, which performs more than 4000 interventional cardiac operations and treats about 800 patients with AMI every year. Patients who may qualify for the study will be screened by a research team consisting of clinicians who are familiar with AMI diagnosis and treatment. The research team consisted of emergency physicians, cardiac intensive care unit physicians, and cardiologists, all of whom are trained in all relevant information about the study. Patients who met the criteria will be actively mobilized to participate in the study from the emergency room to the cardiac intensive care unit or cardiology ward, thereby increasing the enrollment rate.

### Inclusion criteria

Patients eligible for enrollment will be men and women, 18 years of age or older, who are diagnosed with AMI. Diagnostic criteria for AMI were determined according to the Fourth Universal Definition of Myocardial Infarction [14].

A patient with elevated cardiac biomarkers (mainly cTnT) above the 99th percentile of the upper limit of the reference value and who meets at least one of the following conditions is considered to be diagnosed with AMI.

1. Ischemic symptoms.
2. Electrocardiograph has typical manifestations.
3. Echocardiography showed segmental abnormal ventricular wall motion.
4. Intravascular thrombosis confirmed by coronary angiography or autopsy.

### Exclusion criteria

The following are the exclusion criteria:

1. Breast-feeding or pregnant women (pregnancy tests will be performed on women of child-bearing age prior to enrollment)
2. Patients who need probiotics for diarrhea or have used microecological preparations within 1 month
3. Patients with intolerance to microecological preparations
4. Patients with immunodeficiency or who have long-term use of immunosuppressive agents
5. Patients who have stable or unstable angina pectoris that did not fully develop to myocardial infarction

### Withdrawal criteria

Participants will withdraw from the study for any of the following reasons:

1. Patients who experience serious adverse events (AEs) throughout the trial
2. Participants with poor compliance and could not cooperate with clinical examination and follow-up
3. Patients who quit this clinical trial voluntarily

### Informed consent

Prior to enrollment, the trained researcher will introduce the purpose and content of this study to the qualified AMI patients as well as their families. Patients and their families will also be informed of the possible benefits and potential risks. They will have 24 h to consider whether to participate in the trial or not to ensure that patients are entirely voluntary. Furthermore, we will explain to the patients that participating or not participating in the study does not affect their normal treatment. Clues to the participant’s personal information, such as name and hospital number, will be coded instead. The personal information of all participants will always be kept confidential.

### Randomization and allocation

Eligible patients will be randomized in a 1:1 ratio to receive bifidobacteria triple viable capsule or only receive the standard treatment strategy immediately after signing the informed consent form. Randomization will be performed blockwise with variable-sized blocks. The block sizes will not be disclosed to ensure concealment. Random number tables will be generated by a study-independent staff with no other involvement in the study using the SPSS statistical software (SPSS Inc., Chicago, IL, USA, version 25). The researcher will be informed of the treatment allocation plan of the patients after sending basic information about eligible subjects to the staff of the central random system through the Internet.

### Interventions

**Fig. 1:**
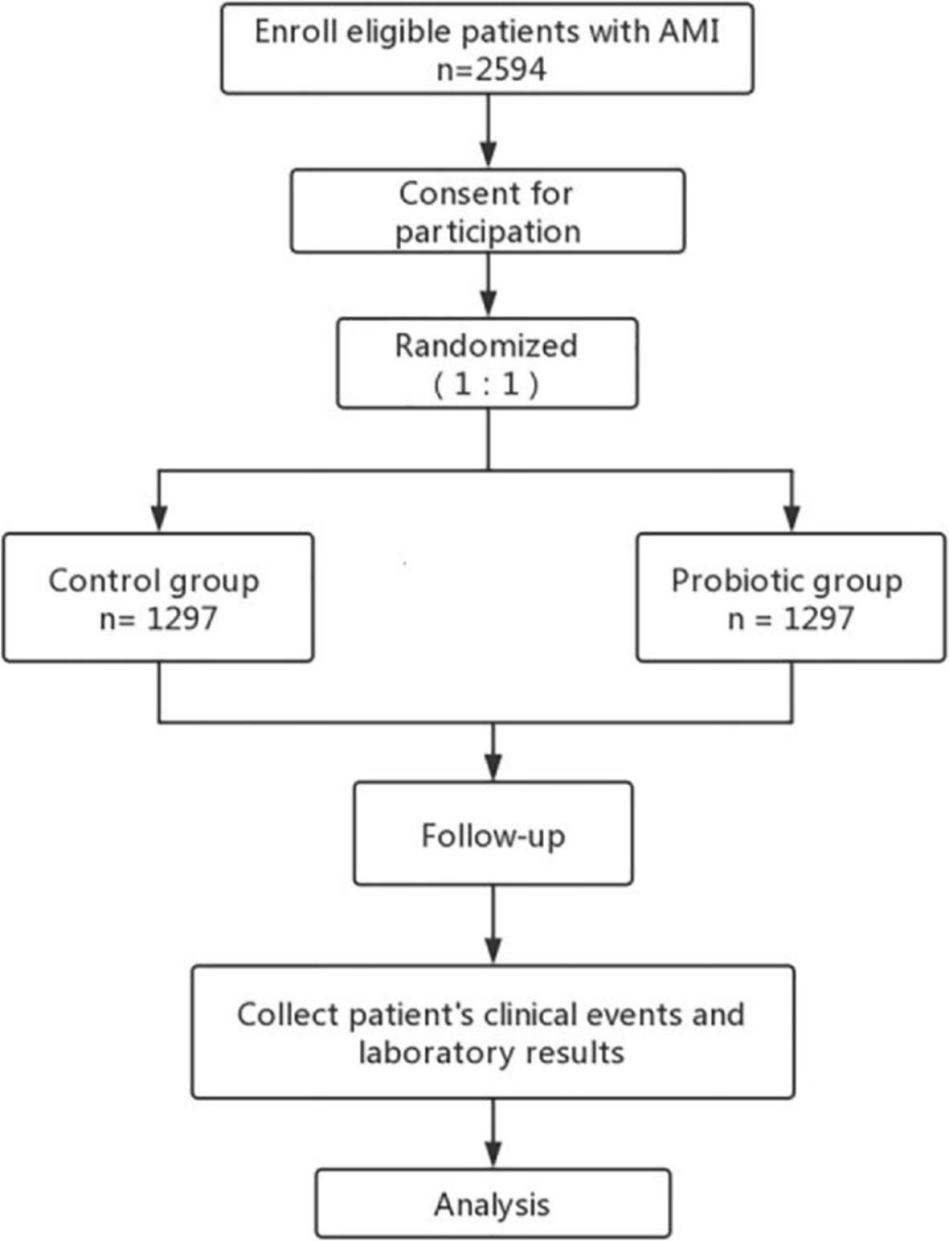
Participant flow diagram

Both groups will receive necessary treatments, such as interventional therapy/thrombolysis and medication for coronary heart disease (including but not limited to dual anti-platelet therapy, AECI/ARB, beta blockers, lipid-lowering drugs). Participants will be randomly assigned in 1:1 to either a probiotic group (bifidobacteria triple live bacteria capsules, 840 mg twice a day) or a control group (without bifidobacteria triple live bacteria capsules) during the hospitalization, for a maximum of 30 days. Each probiotic capsule contains active *Bifidobacterium longum*, *Lactobacillus acidophilu*s, and *Enterococcus faecalis*, with the concentration of each bacterium being 1 × 10^7^ CFU/210 mg. During the study period, patients or their guardians will be instructed to record their daily medication as required to ensure compliance. The drug will be discontinued when patients incur severe adverse reactions or required to withdraw from the trial. A brief overview of the study process is provided in Fig. 1. Bifidobacteria triple live bacteria capsules will be provided by Shanghai Xinyi Pharmaceutical Co., Ltd. (Shanghai, China).

### Blinding

This is an open-label trial. Trial participants, investigators, caregivers, outcome assessors, and data analysts are not blinded to the group assignment.

### Outcome measurements

Hospitalized patients will be followed daily until the occurrence of a primary end-point event or discharge from the hospital. Telephone follow-up will be conducted on the 30th day after admission to understand the survival status of the patients. For patients who have been hospitalized for more than 30 days, end-point events will be collected on day 30 of hospitalization.

#### Primary outcome

The primary outcome will be in-hospital all-cause mortality at 30 days. We will keep track of each patient’s survival through telephone follow-up and the electronic medical record system, and in case of death, the cause of death will be recorded. At the end of the trial, the total number of participants, the number and proportion of deaths in the two groups will be counted, and the in-hospital mortality will be compared between the two groups.

#### Secondary outcome

Secondary outcomes will be clinical complications and laboratory results serve as surrogate end-points for prognosis. The secondary outcomes include the following:

- The incidence of MACE (including heart failure, malignant arrhythmias, cardiogenic shock, cardiogenic death, and recurrent myocardial infarction) during hospitalization or at 30-day follow-up
- The incidence of myocardial infarction-related mechanical complications during hospitalization or at 30-day follow-up
- The difference between the trial groups in left ventricular ejection (LVEF) measured by echocardiography at baseline, 7th day, and 30th day

#### Safety outcome

The safety outcomes will be occurrence of allergies, sepsis, and severe diarrhea/constipation.

### Definition

In this study, MACE is defined as heart failure, malignant arrhythmias, cardiogenic shock, cardiac death, and recurrent myocardial infarction.

- According to the AHA/ACC/HFSA Guidelines for Managing Heart Failure [15], the diagnosis of heart failure is mainly based on clinical manifestations and physical examination: (1) typical clinical manifestations of new-onset heart failure (such as dyspnea, ankle edema, fatigue), (2) moist rales could be heard in more than one-third of the lung fields, (3) chest X-ray showed non-cardiac pulmonary edema or pulmonary obstruction, and (4) abnormal levels of brain natriuretic peptide or B-type natriuretic peptide precursor.
- Malignant arrhythmias are defined as atrial fibrillation/flutter, ventricular tachycardia, and high-degreee atrioventricular block.
- The criteria for cardiogenic shock include less than 90 mmHg systolic blood pressure, continuing for at least 30 min, clinical signs of pulmonary congestion, and organ perfusion for signs of damage to at least one of the following performance: the mental state changes, skin and limbs cold and wet, oliguria, and urinate less than 30 ml per hour.
- Cardiac death is defined as death from any of several cardiac causes.
- Recurring myocardial infarction is defined as another myocardial infarction 28 days after the onset of acute myocardial infarction, with ST-elevation ≥ 1 mm recurs, or new pathogomonic Q waves appear in at least two contiguous leads, especially when accompanied by symptoms of ischemia.
- Myocardial infarction-related mechanical complications include cardiac rupture, ventricular septal rupture, and papillary muscle rupture.

### Follow-up

During the informed consent period, the researcher will have the patient consider whether they can complete the follow-up and then decide whether to participate in the study. In order to facilitate participant retention and complete follow-up, addresses and phone numbers of participants and their families will be collected after consent was obtained. In addition, we will request the physician in charge who is trusted by the patient to assist in the follow-up of the patient. The follow-up period will be divided into two phases, during hospitalization and after discharge. According to statistics, the average hospitalization time of patients with AMI in the First Affiliated Hospital of Shantou University Medical College was 10 days. During hospitalization, the staff will be scheduled to follow up daily, mainly to understand the occurrence of adverse events and the compliance of patients with medication, for a maximum of 30 days. Patients who have been hospitalized for less than 30 days will be followed up by phone or face-to-face on day 30, mainly to check the patient’s survival status and any readmission within 30 days. If there is a readmission within 30 days, further information on the reason for the admission and the presence of adverse cardiovascular events will be obtained. The detailed study schedule is listed in Table 1. Patients who completed the 30-day follow-up were considered to have met the clinical and trial requirements. Patients who discontinued the study drug prematurely will be marked as off-study drug/on study and will follow the same schedule of events as those patients who continue study treatment.

**Table 1:** Timeline of trial procedures and follow-up schedule

Study period															
	Screening	Baseline	Follow-up												
Time point (days)	− 1	0	1	2	3	4	5	6	7	8	9	10	etc	30th	Close-out
Eligibility check	X	X													
Informed consent	X														
Relevant medical history taken	X	X													
Physical examination	X	X	X	X	X	X	X	X	X	X	X	X	X	X	
Full blood count		X							X			X		X	
Lipid levels		X							X			X		X	
Liver and renal function		X							X			X		X	
Myocardial enzymes		X							X			X		X	
C-reactive protein		X							X			X		X	
Pro-BNP		X							X			X		X	
HBALC		X							X			X		X	
Electrocardiogram		X							X			X		X	
Echocardiography		X							X			X		X	
All-cause death		X	X	X	X	X	X	X	X	X	X	X	X	X	X
MACE		X	X	X	X	X	X	X	X	X	X	X	X	X	X
Serious adverse events		X	X	X	X	X	X	X	X	X	X	X	X	X	X

*Pro-BNP* N-terminal pro-brain natriuretic peptide, *MACE* major adverse cardiovascular events

### Sample size

Published data indicate that hospitalization mortality for acute myocardial infarction is 8.9% [3]. Since there are no reports of probiotics reducing mortality in people with myocardial infarction, we conducted a pilot experiment and found that probiotics reduce the risk of death, in people with acute myocardial infarction by 40%. Hence, we predict that treatment with probiotics will reduce mortality to 5.34% with no change in the placebo group. A sample size of 1102 patients in each arm is needed to obtain 90% power to demonstrate a statistically significant difference (two-sided test, alpha = 0.05) between the two treatments. We will increase the sample size to 130 in each group to provide for a 15% dropout rate. A total of 2594 participants (1297 in the probiotic group and 1297 in the control group) will be enrolled. All calculations are done through the PASS software (NCSS, America, version 11), using the log-rank test and Freedman method.

### Data collection and management

At enrollment, we will collect demographic information of participants, including age, height, weight, blood pressure, heart rate, past medical history, smoking history, and laboratory parameters (including leukocytes, neutrophils, creatinine, uric acid, myocardial enzymes, lipid profile, pro-BNP, CRP, LEVF, LVd, LVS) at admission. Besides, death, heart failure, malignant arrhythmias, cardiogenic shock, recurrent myocardial infarction, and other conditions of patients will be recorded during the hospitalization or follow-up at the 30th day. Case report forms (CRFs) will be used to record all participant data. This task will be completed by the researchers, who will also enter the data into an electronic database by duplicate entry using the EpiData software (Denmark, version 3.1). All data will be stored in the scientific research platform of the First Affiliated Hospital of Shantou University Medical College, which is a secure server with limited access and supported for anonymity analysis and review.

### Statistical and analytical plans

The analyses will adhere to intention-to-treat (ITT) principles. The ITT population included eligible patients who had been randomly assigned to participate in the study, regardless of whether any patients were taking a trial drug. Missing observations are accounted for using the predictive mean matching (PMM) method. Descriptive statistics will be used to analyze the results. Continuous data, such as age, height, and weight, will be presented as mean ± SD. Categorical data such as all-cause death, previous disease history, and smoking history will be expressed as frequency and percentage of patients in each category. Comparison baselines will be obtained using an independent sample *t* test. For the primary outcome, the chi-square test will be used to compare the all-cause mortality in both arms. Secondary outcomes will be assessed by the chi-square test for event rate, and the paired *T* test for quantitative secondary endpoints to compare both treatment arms. In addition, Kaplan–Meier curves will be calculated for visualizing the cumulative events in both groups during hospitalization. A *p*-value of less than 0.05 (*p* < 0.05) will be considered statistically significant. SPSS (25.0) will be used for statistical analysis.

The efficacy and safety of probiotics in the treatment of acute myocardial infarction are unknown. The purpose of interim analyses is to enable early detection of trial safety, early detection and termination of the study in the event of safety problems, and early detection of efficacy, which can be halted for efficacy if the trial is effective and has satisfied prespecified criteria. For interim analysis, we will do a group sequential statistical design with 2 interim analysis when we collect 33% (856) and 67% (1815) of the analyzable patients. The interim analysis will be performed using the Pocock method. Each analysis, including the final analysis, will be set at the same significance level alpha = 0.022 to maintain an overall significance level of 0.05. An independent Data Monitoring Committee supported by an independent, unblinded statistical center will regularly review safety data as well as the interim efficacy analysis.

### Monitoring

This study will be performed following the approved protocol. The trial will be monitored by an independent data and safety monitoring committee. This committee is chaired by five independent researchers who are not involved in trial operations and have no conflict of interest with this study. The data and safety monitoring committee will review and interpret the data generated from the study in order to ensure the safety of the participants and the integrity of the research data.

### Serious adverse events

In general, bifidobacterium triple viable capsules within the specification dosage scope are safe and will not produce serious adverse reactions. However, adverse reactions such as allergy, abdominal distension, diarrhea, constipation, and more severe bacteremia may occur. All unexpected serious adverse events will be reported immediately to the Medicines and Healthcare Products Regulatory Agency. Any adverse events will be treated by the physician according to usual practice, and we will also follow the patient until symptom resolution or treatment discontinuation. The cost of adverse reactions will be borne by the project. Adverse events and serious adverse events will be assessed for degree, severity, and causality with the intervention or other possible treatment and recorded in the CRFs.

### Auditing and amendment

All proposed protocol changes will be documented in the protocol amendments and submitted for the ethics committee and regulatory agencies’ approval before implementation.

### Patient and public involvement

No patients or members of the public participated in the conception of our study. However, the results of the study will be published in the appropriate journal after complete data analysis.

## Discussion

This is an open-label, randomized, controlled, superiority clinical trial designed to evaluate the effect of probiotic supplementation on in-hospital mortality in patients with AMI. Disturbance of intestinal flora and bacterial translocation play an integral role in the occurrence and development of AMI, especially in the initial stage after infarction, which is critical for survival [16]. We suppose to regulate intestinal flora through probiotics, reduce the production of harmful metabolites of intestinal flora, repair intestinal barrier, and reduce systemic inflammation, so as to control the further development of acute myocardial infarction and improve survival rate.

In recent years, intestinal flora has been revealed to have a close relationship with myocardial infarction. The main mechanisms include the direct effect of microbial metabolites on atherosclerosis and thrombosis [17], endotoxemia, and systemic inflammation following intestinal microbial translocation indirectly exacerbate ventricular remodeling after myocardial infarction [18]. Besides, TMAO, a product of intestinal flora metabolism [19], have been confirmed as an independent predictor of atherosclerotic burden and long-term mortality in patients with coronary artery disease and a prognostic marker of MACE in chronic heart failure patients after MI [20–22]. Regulation of intestinal flora is a promising new target for the treatment of myocardial infarction. Probiotic administration has been considered as an effective and safe approach for the treatment of cardiometabolic diseases such as hypertension, diabetes, and hyperlipidemia [23]. In an animal study, probiotics supplementation in MI model mice can reduce the size of myocardial infarction and inflammatory infiltration of myocardial cells [8]. Moludi et al. showed that probiotics reduce TMAO levels and reduce ventricular remodeling in MI patients [24]. From the perspective of heart failure, studies have shown that probiotic supplementation can help improve ventricular systolic function and quality of life in patients with heart failure [25]. Furthermore, we are of the opinion that probiotics may also play a positive role in the treatment of patients with MI in terms of defecation. Although probiotics are not routinely recommended in the guidelines, it is mentioned in the consensus of Chinese myocardial infarction treatment experts that AMI patients should keep their bowels open [26]. Clinically, forced defecation is a common cause of heart failure after myocardial infarction. Bifidobacteria have been shown to have a laxative function [27] and may play a role in reducing the incidence of adverse events after AMI.

The above results indicate that probiotics may reduce the incidence of cardiovascular adverse events and mortality by reducing myocardial infarction area expansion and reducing ventricular remodeling. However, to our knowledge, there are no data to report whether probiotics can reduce mortality or adverse events in patients with myocardial infarction. Therefore, we will further demonstrate the effect of probiotics on the prognosis of patients with acute myocardial infarction through an RCT by evaluating in-hospital mortality and incidence of MACE (acute heart failure, malignant arrhythmia, cardiogenic shock) and myocardial infarction-related mechanical complications in patients.

Our study has some limitations. First, this is a single-center clinical trial, and the results are not representative of all patients with AMI. Secondly, bifidobacterium triple live capsule is a compound microecological agent containing three kinds of bacteria. Although it can help regulate intestinal flora, we cannot explain which bacteria are effective. Furthermore, we do not conduct microflora analysis on the blood and feces of patients in this study. If the initial results are positive, we plan to analyze blood and feces samples from AMI patients on the first day of hospitalization and after treatment to further confirm the influence of probiotics on the intestinal microflora of AMI patients.

In summary, the primary therapeutic goal of modern cardiology is to reduce inflammation and myocardial necrosis and to enhance cardiac repair after myocardial infarction [28]. If the results of this investigation show are effective, it will provide strong evidence of the value of probiotics as an intervention to reduce mortality and improve outcomes in patients with AMI.

## Trial status

The version of this protocol is 2.0 (date 20,210,321). Recruitment began in October 2020 and is estimated to be completed by October 2025. Currently, the trial is in the process of recruiting patients.

## Supplementary Information

**Additional file 1.**

**Additional file 2.**

## Data Availability

The datasets used and/or analyzed during the current study are available from the corresponding author upon reasonable request. The trial data will be publicly available for publication in international peer-reviewed journals, regardless of whether the results are positive or negative.

## Abbreviations

ACS: Acute coronary syndrome
AEs: Serious adverse events
AMI: Acute myocardial infarction
CFU: Colony-forming units
LVEF: Left ventricular ejection fraction
MACE: Major adverse cardiovascular events
PCI: Percutaneous coronary intervention
RCT: Randomized controlled trial
TMAO: Trimethylamine oxide
