# Supplementary material for: Effect of probiotic supplementation on in-hospital mortality in patients with acute myocardial infarction: a study protocol for an open-label, randomized, controlled, superiority clinical trial
Source: Trials. 2023 Jun 24;24:429. doi: 10.1186/s13063-023-07443-5 (PMC10290309; doi:10.1186/s13063-023-07443-5)
Supplement: Supplementary file 1 — Additional file 1. [file 13063_2023_7443_MOESM1_ESM.docx]

Statement

This study has been registered in Chinese Clinical Trials Registry(www.chictr.org.cn). All trial registration items from the WHO trial registry data set can be found in the protocol.

Supplementary table1: Trial registry data.

| **Data category** | **Imformation** |
| --- | --- |
| Primary registry and trial indentifying number | ChiCTR2000038797 |
| Date of Registration | 2020-10-02 |
| Primary sponsor | The first Affiliated Hospital of Shantou University Medical College |
| Public title | Effects of probiotics on in-hospital mortality in patients with acute myocardial infarction: a randomized, open-label, superiority clinical trial |
| Scientific title | Effects of probiotics on in-hospital mortality in patients with acute myocardial infarction: a randomized, open-label, superiority clinical trial |
| Approved No. of ethic committee | B-2020-165-FS |
| Name of the ethic committee | Ethics Committee of the First Affiliated Hospital of Shantou University Medical College |
| Countries of recruitment | China |
| Interventions | Experimental group:bifidobacterium triple viable capsule 840mg bid. |
|  | Control group:blank control. |
| Inclusion criteria and exclusion criteria | Inclusion criteria: Patients eligible for enrollment will be men and women, 18 years of age or older, who are diagnosed as AMI. |
|  | Exclusion criteria: Breast-feeding or pregnant women/have used microecological preparations within one month/intolerance to microecological preparations/immunodeficiency or have long-term use of immunosuppressive agents. |
| Study type | Interventional |
| Date of first enrolment | 2020.10.3 |
| Target sample size | 2597 |
| Recruitment status | Recruiting |
| Primary outcome(s) | In-hospital mortality |
| Key second outcome(s) | Major adverse cardiovascular events (MACE) |
